# Diagnostic Reliability of Leptomeningeal Disease Using Magnetic Resonance Imaging

**DOI:** 10.7759/cureus.4416

**Published:** 2019-04-09

**Authors:** Philippe Harris, Ange Diouf, François Guilbert, Fatima Ameur, Laurent Letourneau-Guillon, Cynthia Ménard, Laura Masucci, Manon Bélair, David Roberge

**Affiliations:** 1 Miscellaneous, Laval University, Montréal, CAN; 2 Radiology, University of Montreal Health Centre, Montréal, CAN; 3 Radiology, University Hospital of Lyon, Lyon, FRA; 4 Radiation Oncology, University of Montreal Health Centre, Montréal, CAN

**Keywords:** leptomeningeal disease, neuroimaging, inter-observer agreement, mri, radiation oncology, radiology, brain metastasis

## Abstract

Brain metastases are seen in 20%-50% of patients with metastatic solid tumors. On the other hand, leptomeningeal disease (LMD) occurs more rarely. The gold standard for the diagnosis of LMD is serial cerebrospinal fluid (CSF) analyses, although in daily practice, the diagnosis of LMD is often made by neuroimaging. Leptomeningeal metastases (LM) have been a relative contra-indication to radiosurgery. It can be noted that focal LMD can be difficult to distinguish from a superficially located/cortical-based brain metastasis which is not a contra-indication for radiosurgery. Hence, justifying the need of a reliable diagnosis method.

The goal of this study was to determine the inter-observer reliability of contrast-enhanced magnetic resonance imaging (gdMRI) in the differentiation of focal cortical-based metastases from leptomeningeal spread.

This is a retrospective review of a prospectively collected database of patients with brain metastases. A total of 42 cases with superficial lesions were selected for review. Additionally, eight control cases demonstrating deep and/or white-matter based lesions were included in the study.

Three neuroradiologists and three radiation oncologists were asked to review each study and score the presence of LM. Inter-observer agreement was calculated using group-derived agreement coefficients (Gwet’s AC1 and Gwet's AC2). Pair-wise inter-observer agreement coefficients never reached substantial values for trichotomized outcomes (LMD, non-LMD or indeterminate) but did reach a substantial value in a minority of cases for dichotomised outcomes (LMD or non-LMD). The control subgroup analysis revealed substantial agreement between most pairs for both trichotomized and dichotomised outcomes.

We observed low inter-observer agreement amongst specialists for the diagnosis of focal LMD by gdMRI. Neuroimaging should not be relied upon to make treatment decisions, notably to deny patients radiosurgery.

## Introduction

Brain metastases occur in 20%-50% of patients with advanced cancer. Leptomeningeal disease (LMD) is diagnosed in 5%-10% of patients with otherwise metastatic cancer, although rates approaching 20% have been reported in autopsy series [1-2]. The gold standard for the diagnosis of LMD is serial cerebrospinal fluid (CSF) analyses, which has a sensitivity of approximately 55% on the first CSF examination and reaches up to 85% sensitivity when done thrice - while maintaining a theoretical specificity of 100% [3-4]. The presence of malignant cells in a sample of CSF, cranial nerve palsies or widespread meningeal enhancement (particularly in the cerebellar folia) is convincing evidence of a process that is not amenable to focal treatment. On the other hand, nodular lesions described as being of leptomeningeal origin can cause more therapeutic ambiguity. In current practice, diagnosis of LMD is also often made without CSF analysis, using gadolinium-enhanced magnetic resonance imaging (gdMRI) of the brain and/or spinal cord, which is reported to have a sensitivity and specificity of approximately 75% [5-6]. LMD thus diagnosed can represent a relative contra-indication to stereotactic radiosurgery.

The goal of this study is to characterize the inter-observer reliability of gdMRI to differentiate focal cortical-based metastases from focal LMD in the context of known metastatic disease.

## Materials and methods

This is a retrospective study of cerebral magnetic resonance imaging (MRI) studies of patients suspected to have focal leptomeningeal metastases (LM). All patients included in this study underwent radiosurgery between 2009 and 2013. The study protocol was approved by our institutional ethics review board.

We completed a retrospective review of a prospectively collected database of 438 patients with known primary disease and neurological symptoms referred for evaluation to our tertiary care center radiation-oncology clinic between May 2009 and June 2013. Of these patients, 174 had a reviewable planning MRI prior to intervention in radiation-oncology. These pre-intervention studies were screened randomly by a single author (FG, neuroradiologist with 15 years of experience) until 42 cases were selected for review, while blinded to the original imaging report. The sole inclusion criteria was the presence of one or more enhancing cortical-based lesions. Cases demonstrating widespread leptomeningeal or cranial nerve enhancement, as well as cases with previous radiosurgery or surgery were excluded from the study, as were cases with suboptimal study quality. Additionally, eight control cases were included in the study, consisting of eight patients demonstrating only deep and/or white-matter based lesions.

Readers included three neuroradiologists (two senior staff members (5-18 years of experience) and a neuro-radiology fellowship trainee) and three radiation oncologists with active radiosurgery practices (5-15 years of experience).

All MRIs studies were independently reviewed by each of our six examiners using our institution’s Picture Archiving and Communication System (PACS), and were assigned to one of three diagnostic categories (LMD, non-LMD, indeterminate). To establish a diagnosis per specialty, cases who received the same diagnostic label by two readers were assigned to that category, while cases who received three different diagnoses were re-evaluated by the three specialists to establish a consensus diagnosis.

In addition to analysing the data using a ternary scale (LMD, non-LMD, indeterminate), statistical analyses were also performed using a binary scale, in which cases assigned to the “indeterminate” category were clustered with cases assigned to the “non-LMD” category, based on the assumption that patients without a firm diagnosis of LMD would be treated in a similar fashion in regards to radiotherapy.

Inter-rater coefficients were calculated between each pair of readers using Gwet’s AC1 and AC2, for dichotomized and trichotomized ratings respectively, due to their more robust nature, allowing inter-observer analyses on data demonstrating a low prevalence index and presence of constants, notably in the controls. Gwet’s AC1 and AC2 was also used to derive group inter-rater coefficients amongst the entire cohort, as well as subgroups (amongst radiologists, radio-oncologists and between the two specialties, using the consensus agreements described above).

Agreement coefficients are interpreted using the highest benchmark level that is associated with the smallest cumulative membership probability (CMP) that exceeds 95% [7]. The specific benchmark was the one described by Landis and Koch [8] (< 0, poor agreement; 0-0.20, slight agreement; 0.21-0.40, fair agreement; 0.41-0.60, moderate agreement; 0.61-0.80, substantial agreement; and 0.81-1.00, almost perfect agreement).

## Results

**Figure 1:**
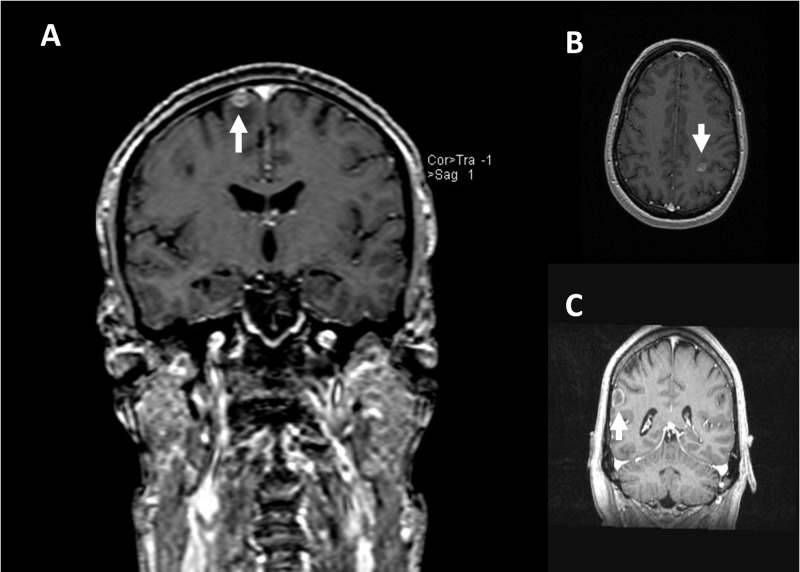
Patient 1 magnetic resonance imaging (MRI) causing division between readers T1-weighted gadolinium enhanced images of candidate lesions causing division between readers (A: Coronal view, B: Axial view, C: Coronal view).

**Figure 2:**
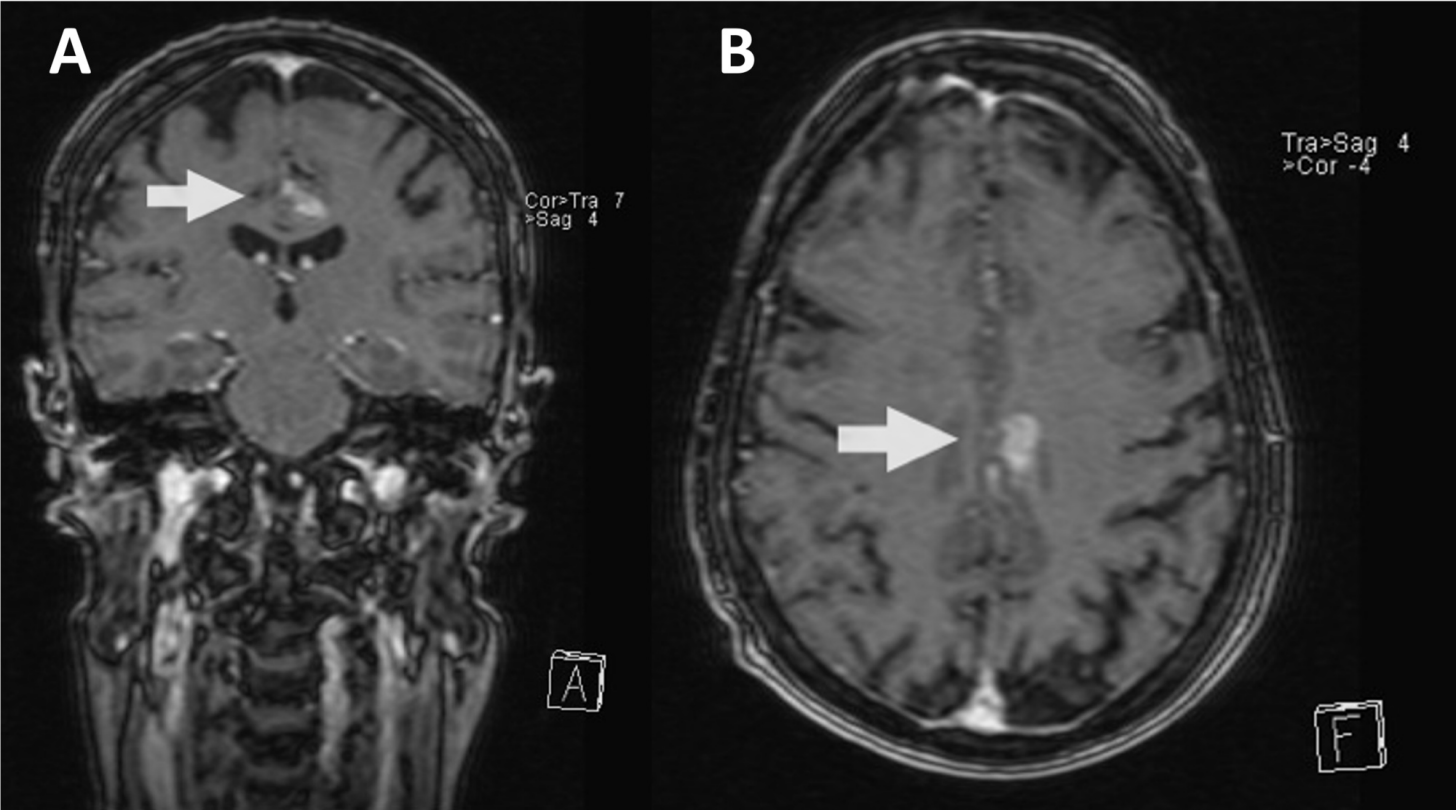
Patient 2 magnetic resonance imaging (MRI) causing division between readers T1-weighted gadolinium enhanced images of candidate lesions causing division between readers (A: Coronal view, B: Axial view).

While some cases achieved an agreement by all readers, some other cases presented more ambiguous lesions thus causing division amongst readers (Figures 1-2).

In trichotomized ratings, only one out of the 42 cases and only three out of the eight controls were assigned to the same diagnostic category by all six raters. All other studies were assigned to two different diagnostic categories (out of three) by at least two raters, with variable agreement amongst the rest of the raters. In dichotomized ratings, these numbers rose to nine out of 42 cases and four out of eight controls. In both trichotomized and dichotomized ratings, only the non-leptomeningeal category was assigned by all six raters. No consensus was obtained within the leptomeningeal category.

Ternary inter-rater agreement coefficients amongst pairs of raters ranged from poor (AC1 = -0.16; 95% CI: -0.50, 0.19) to fair (AC1 = 0.56; 95% CI: 0.34, 0.79), while they ranged from poor (AC1 = 0.18; 95% CI: 0, 0.49) to substantial (AC1 = 0.80; 95% CI: 0.66, 0.95) in binary analyses. Using cumulative membership probabilities, only one inter-rater coefficient reached a substantial value for the dichotomized ratings, while none did so for the trichotomized ratings.

In the control subgroup analysis, inter-rater agreement coefficients amongst pairs of raters ranged from poor (AC2 = 0.25; 95% CI: -0.55, 1.06) to almost perfect (AC2 = 0.94; 95% CI: 0.82, 1.06) for trichotomized ratings, while they ranged from poor (AC1 = 0.34; 95% CI: -0.37, 1.058) to perfect agreement (AC1 = 1.00) for dichotomized ratings. Out of 15 pairs of raters, seven demonstrated an inter-rater agreement coefficient > 0.61 for trichotomized ratings; 11 for dichotomized ratings. 

Interrater agreement coefficient amongst the entire cohort was slight (AC2 = 0.19; 95% CI: 0.01, 0.38) for trichotomized ratings and fair (AC1 = 0.43; 95% CI: 0.28, 0.59) for dichotomized ratings. By specialty, inter-rater agreement coefficients amongst radiologist was slight (AC2 = 0.35; 95% CI: 0.14, 0.57) for trichotomized ratings and moderate (AC1 = 0.66; 95% CI: 0.50, 0.82) for dichotomized ratings, while radio-oncologists demonstrated poor (AC2 = 0.17; 95% CI: -0.05, 0.40) and slight agreement (AC1 = 0.34; 95% CI: 0.13, 0.54) for trichotomized and dichotomized ratings, respectively (Table 1). Agreement between the two specialties was poor for both trichotomized (AC2 = -0.13; 95% CI: -0.34, 0.084) and dichotomized (AC1 = 0.02; 95% CI: -0.150, 0.193) ratings (Table 2). For controls, group-derived inter-rater agreement coefficients reached a substantial value for the entire cohort, for both trichotomized (AC2 = 0.60; 95% CI: 0.18, 1.02) and dichotomized (AC1 = 0.68; 95% CI: 0.32, 1.04) ratings.

**Table 1:** Group derived inter-rater agreement coefficients Gwet's AC1: Gwet Inter-rater agreement coefficient; Gwet's AC2: Gwet Inter-rater agreement coefficient with quadratic weighting; StdErr: Standard error of agreement coefficients; 95% C.I.: 95% Confidence interval; CMP: Interpretation of agreement coefficients based on benchmark values described by Landis and Koch, using cumulative membership probabilities (CMPs).

			Coefficient	Inference/Subjects		
				StdErr	95% C.I.	Interpretation using CMPs
Radiologists (n=3)	Ternary	Gwet's AC2	0,35452	0,10929	0.135 to 0.574	Slight
	Binary	Gwet's AC1	0,66208	0,07901	0.503 to 0.821	Moderate
Radio-oncologists (n=3)	Ternary	Gwet's AC2	0,17477	0,10993	-0.046 to 0.396	Poor
	Binary	Gwet's AC1	0,33685	0,10196	0.132 to 0.542	Slight
Cohort (n=6)	Ternary	Gwet's AC2	0,19222	0,09073	0.011 to 0.375	Slight
	Binary	Gwet's AC1	0,43436	0,07789	0.278 to 0.591	Fair

**Table 2:** Group derived inter-specialty agreements coefficients Gwet's AC1: Gwet Inter-rater agreement coefficient; Gwet's AC2: Gwet Inter-rater agreement coefficient with quadratic weighting; StdErr: Standard error of agreement coefficients; 95% C.I.: 95% Confidence interval; CMP: Interpretation of agreement coefficients based on benchmark values described by Landis and Koch, using cumulative membership probabilities (CMPs).

			Coefficient	Inference/Subjects		
				StdErr	95% C.I.	Interpretation using CMPs
Radiologists (n=3) vs Radio-oncologists (n=3)	Ternary	Gwet's AC2	-0,12700	0,10546	-0.338 to 0.084	Poor
	Binary	Gwet's AC1	0,02135	0,08579	-0.150 to 0.193	Poor

## Discussion

Cortical-based metastases are difficult to differentiate radiologically from focal leptomeningeal carcinomatosis. Our study demonstrates significant variability in the interpretation of studies demonstrating cortical-based enhancing disease, both amongst clinicians and radiologists.

However, inter-rater agreement was nonetheless higher amongst neuro-radiologists (moderate) than radio-oncologists (slight), suggesting increased reliability of certain radiological signs, best evaluated with radiological expertise.

LMD remains a clinical diagnosis, based on clinical symptoms, imaging, and cerebrospinal fluid analysis. Presence of CSF cytology remains the gold standard, with 60% sensitivity on initial puncture, 80% total sensitivity on second puncture and 2%-5% sensitivity increase per following repeated collection [9]. Most diagnostic algorithms recommend initial evaluation with a CSF profile, CSF cytology and gdMRI of the neuroaxis, with at least one additional lumbar puncture if initial CSF cytology is negative [9-12]. Meanwhile, the frequency of LM-related MRI abnormalities for solid tumors on neuroaxis imaging varies from 56% to 65%, depending on the primary [13]. Although in some cases, the constellation of clinical symptoms of LMD combined with bulky LMD on neuroaxis imaging may obviate the need for lumbar puncture, our study shows that, in more mitigated cases, focal cortical-based metastases are difficult to differentiate from focal LMD [3-4,6]. In such cases, we advocate that imaging findings should be correlated by CSF analysis, serial of need be, before LMD can be diagnosed, in large part because of the implication for treatment, most notably radiotherapy.

Although there is no universally accepted standard treatment for LMD, whole-brain radiotherapy (WBRT) is often considered in this patient population. While some cases can benefit from WBRT, this treatment does expose healthy brain tissue to high doses of radiations [14]. Adverse effects of cerebral radiation therapy may include neurocognitive disorders, radionecrosis and, rarely, radiation-associated meningiomas (RAM) [15]. Alternatively, stereotactic surgery (SRS) achieve high rate of local control while diminishing radiation exposure to the rest of the brain [2].

Ross et al. outlined the importance of a clearly established diagnostic before exposing patients to SRS or WBRT, as the implications of misdiagnosis might either be radiation overexposure or missed tumors [16]. Flickinger et al. argue that imaging alone represents an accepted diagnostic tool although they report a 2.3% chance of misdiagnosis, when imaging is not confirmed by CSF analysis [17]. Alternatively, Wolf et al. suggest that the use of SRS might successfully treat focal LMD and delay WBRT for some patients [2]. Consequently, radiosurgical approach has been used as a treatment for focal LMD [2,18].

Therefore, because LMD is often difficult to differentiate from cortical based metastases and because initial focal radiotherapy may delay whole-brain radiotherapy, we argue that patients with a known solid primary neoplasm and equivocal imaging findings for LMD should still be considered for stereotactic surgery initially.

## Conclusions

The diagnosis of nodular LMD as opposed to cortical-based brain metastases on gadolinium-enhanced brain MRI demonstrates low inter-observer agreement amongst specialists, both radiologist and radio-oncologists. If imaging findings may alter the treatment approach, they should at least be confirmed by serial CSF analysis.
